# A dynamic journey of comprehensive school health policy implementation in response to the COVID-19 pandemic in Lombok, Indonesia

**DOI:** 10.1186/s41182-025-00690-z

**Published:** 2025-02-20

**Authors:** Cut Warnaini, Abiyyu Didar Haq, Hamsu Kadriyan, Fumiko Shibuya, Jun Kobayashi

**Affiliations:** 1 Faculty of Medicine and Health Sciences, University of Mataram, Jl. Pendidikan No. 37, Nusa Tenggara Barat, 83125 Mataram, Indonesia; 2 Department of Global Health, Graduate School of Health Sciences, University of the Ryukyus, 207 Uehara, Nishihara, Okinawa 903-0215 Japan; 3 Japanese Consortium for Global School Health Research, Okinawa, 207 Uehara, Nakagami-Gun, Nishihara, Okinawa 903-0215 Japan

**Keywords:** Comprehensive school health, Primary schools, Secondary schools, COVID-19 pandemic, Collaboration, Community engagement

## Abstract

**Background:**

Coronavirus disease 2019 (COVID-19) pandemic in March 2020 led to new restrictive policies in several countries, including Indonesia. The comprehensive school health (CSH) framework provides overall guidance for interventions most effective in achieving specific outcomes related to health, nutrition, and education, and creating CSH programmes that account for the main contextual variations in Indonesian communities and schools is important. This study aimed to clarify how school health-related policies made before and after the COVID-19 pandemic responded to COVID-19 control measures on Lombok Island, Indonesia.

**Method:**

This was a qualitative observational analytic study. Researchers reviewed and analysed school health policy, held separate confirmation discussions and interviews with stakeholders and key informants, and observed policy implementation at public and private schools in Mataram.

**Results:**

The analysis found weaknesses and opportunities. Weakness included lack of guidelines, comprehensive planning, inconsistent hierarchy of roles and responsibilities, and social and cultural barriers. Opportunities included the importance of funding for consistent CSH implementation, monitoring and evaluation system, implementation between public and private schools, and decentralisation. Positive findings included CSH policy integration into teaching and learning activities, regular healthy school competition program, teachers as role models, existing coordination and distribution of responsibilities between relevant stakeholders, and authorising schools to make needed adjustments. Factors influencing school health efforts included curriculum, school organisation, personal ethos, and healthcare provider partnerships. To effectively promote school health efforts, schools must assess existing health problems within the school environment and surroundings, organisational structure and capabilities including knowledge and skills, commitment, and leadership aspects.

**Conclusion:**

The COVID-19 pandemic has prompted the implementation of CSH policies in schools of Lombok Island, demonstrating flexibility and dedication to student welfare. Despite confusion due to changing regulations, collaboration with local health organisations and community support has resulted in effective policy implementation.

## Background

On 12 March 2020, the World Health Organization (WHO) classified coronavirus disease 2019 (COVID-19) as a pandemic. As both direct and indirect encounters could result in the propagation of severe acute respiratory syndrome coronavirus 2 (SARS-CoV-2), isolation, lockdown, and travel restrictions were immediately implemented to stop the SARS-CoV-2 infection from spreading. While these restrictions were thought to have been beneficial in reducing the likelihood of transmission, the impact of other factors, such as the socioeconomic and educational ramifications, were heavily underlooked and turned out to be significant [1, 2]. An additional risk resulted from mutation of the virus, which amplifies its capacity to spread and/or impact the immune system. The elevated COVID-19 mortality in 2021 and that caused by the Omicron version in early 2022 were a result of the Delta variant (B.1.617.2). Some countries, including Indonesia, implemented new restriction rules in response to the appearance of these novel varieties, albeit the specifics of each country’s implementation varied greatly [3]. Schools, extracurricular activities, and childcare/daycare facilities had already been closed down during this time, but some of these programmes were beginning to be reopened in phases. Unfortunately, the intention to reopen schools was threatened by the spread of new SARS-CoV-2 varieties, which also affected the economy and trading activities that had been gradually improving before and throughout the middle of 2021. World Health Organization (WHO) predict that the other pandemic will occur in the future due to the climate changes, the pollution impact, and the used of hazard substances. The latest pandemic that occurs in 2024 was monkeypox.

The COVID-19 pandemic has impacted around 11 billion schools, according to UNESCO. Closing schools is a policy put in place to reduce the possibility of viruses spreading through in-person teaching and learning activities [4]. To address this issue and enable children to continue learning throughout the epidemic, modifications were made to the teaching and learning processes. Remote learning and e-learning are two alternatives used in implementing the teaching and learning processes [5]. These teaching strategies mostly rely on digital media, the internet, and computers and smartphones for interaction, communication, and completion of assignments [6].

Globally, school health has expanded quickly since 2000, with Southeast Asian nations—including Indonesia—reported to be the most advanced in terms of developing and implementing policies. The School Health Unit or Usaha Kesehatan Sekolah (UKS) was established in 1959 as the school health system in Indonesia which functions as the extension of public health centre or Pusat Kesehatan Masyarakat (Puskesmas) in the school area consisted of students and teachers that has been chosen to promote healthy school environment and being guided and trained by health staff from said public health centres. This was a comprehensive school health (CSH) strategy akin to the Health Promoting Schools model that is currently being used globally. It encompassed not just health services and health education but also the building of a healthy school environment.

Sanitation and hygiene are important components of CSH; they continue to be major causes of undernourishment and may impact overall student’s health and well-being, and they may cause obstruction to access to high-quality education, especially in rural regions such as Lombok, West Nusa Tenggara, Indonesia. Disparities between urban and rural areas in access to clean water and sanitary facilities expose ongoing injustices. It is established that rural schools have lower rates of access to sufficient sanitation and clean water than do urban schools. To enhance sanitation and provide access to clean drinking water, more research is required in Lombok. This problem is made worse by the fact that the island’s clean water sources have shrunk by 19.03% since the 2018 earthquake [7]. The CSH framework offers broad recommendations for the kind of intervention that works best to achieve particular goals pertaining to education, nutrition, and health. To make implementation of CSH strategies in current settings as simple and large scale as possible, it is crucial to develop a set of programmes that take into consideration the primary contextual variables seen in Indonesian communities and schools.

This study aimed to clarify how school health-related policies made before and after the pandemic of the new coronavirus infection responded to the control of COVID-19 on Lombok Island, Indonesia. With 38 provinces, Indonesia is among the most decentralised nations. The governance ability of local administrations in the execution of health and education policies also has a major influence on implementation at the school level when the country is moderately sized or larger [8]. Therefore, this study also aimed to investigate the impact of decentralisation on policy execution in Lombok, West Nusa Tenggara Province, Indonesia. For this reason, the study was conducted more than 1000 km away from Jakarta, the country’s capital. This study sought to shed light on the dynamics of Indonesia’s CSH policy to create recommendations for preparedness before an emerging infectious disease pandemic occurs and its ability to adapt during the seismic shift caused by a pandemic.

## Methods

### Study design

The multiple case study design of this study used document reviews and key informant interviews to determine the factors influencing the implementation of school health. A case study examines a current phenomenon in its present context and may include one or more cases to compare a different or similar circumstance. This study focused on the three phases of school health policy and its implementation: Phases #1 and #2, before and during the COVID-19 pandemic, respectively, and Phase #3, after the COVID-19 pandemic. These phases were categorised based on the epidemic timeframe of the SARS-CoV variant. In the conceptual framework, Phase 1 corresponds to the establishment of CSH in Indonesia before the pandemic of the COVID-19, Phase 2 to the early-phase response that included the Alpha and Delta varieties, and Phase 3 to the chronic phase reaction and recovery phase, which included the Omicron version. The document reviews and key informant interviews were used to triangulate the potential convergence of the data acquired from various data sources and ascertain the consistency of the findings across each phase.

A group of study respondents were chosen based on their professional backgrounds in education fields. Group and individual interview respondents were policy-making officials, implementing officials, and also those affected by the school health policy. Those were officials/representatives from the Local Health Office, Local Education Office, Local Religious Affairs Office, local Community/Public Health Center, as well as representatives from local public and private schools. The inclusion of representatives from Local Religious Affairs Office due to Indonesian Public Schools consisted of two types, general public school and Islamic public school (madrasah). This step was considered necessary because we wanted a comprehensive exploration of the school health policy implementation. The respondents were invited to two separate confirmation workshops held in January and May 2022 in which two heads of higher secondary schools were invited. These sessions followed by individual interviews to further confirm the implementation of School Health policy at their respective education institutions. To minimise selection bias as well as to assure confirmability, four targeted-interview respondents from different education levels and groups were chosen: two heads of public primary schools, one head of private primary school, and one head of private lower secondary school.

### Document review

We reviewed the regulations that national and regional leaders had signed. Officially certified web databases controlled by the Indonesian government were used to compile national-level regulatory documents provided by the Presidential Office, the Ministry of Education, and a joint agreement between four ministers. Provincial Education Office and Provincial Health Office personnel provided the local-level regulatory materials. The study team wrote all of the materials in Indonesian and translated them into English. The oldest published records in the search were from 2003, whereas the most recent were published in 2021. The 2003 paper was included because the research team wanted to locate the first public account of the establishment of the UKS.

Information on the foundation of the policies, the procedure for implementing them, and the people involved in creating and conducting the policies are all included in the evaluated documents. In total, 53 papers—11 legal documents, 29 regulatory documents, 4 policy documents, 8 guidelines, and 1 development plan—were identified for the review process. Subsequently, the results were verified through direct observation, discussion sessions for confirmation with relevant stakeholders, and interviews with key informants.

### Confirmation with stakeholders and key informants

We held multiple one-on-one interviews and two group conversations with relevant stakeholders. A modified interview guide designed to understand and grasp the entire process of implementing school health policy was used to facilitate the conversations. The interview topic guide includes questions about implementation strategies, factors that help and hinder implementation, current initiatives and their results, participants and their roles, cooperation with outside organisations, the impact of the ongoing pandemic, and potential future plans.

### School observation

The research team conducted a direct school observation in reference to the modified interview guide used in this study. This activity was undertaken to determine the aspects of the school health policy that were and were not implemented and the participants’ roles and difficulties that they faced. We observed the children and school staff adhering to physical distancing rules and properly implementing health protocols, such as body temperature checkpoints and use of personal protective equipment. Additionally, we examined the classroom, cafeteria or canteen, the library, the restrooms with handwashing stations, the prayer room, the gardens and yards, and waste bins, among others.

### Key informant interviews

Four heads of schools participated in one-on-one interviews with the research team which was led by the first author. The educational institutions in question were located in Mataram City and consisted of one lower secondary school (private) and three primary schools (two public and one private). A group discussion session with two heads of two higher secondary schools was also held by the research team. The purpose of this work was to investigate how each school implemented their school health policy and the difficulties they encountered, particularly during the pandemic. The research team used a previously produced topic guide as a reference when conducting the interview.

### Data analysis

We conducted a comprehensive review of laws, national and regional regulations, and health policies in schools during the COVID-19 pandemic. Additionally, we examined policies and regulations related to COVID-19, hygiene, food and nutrition, mental health, and education for sustainable development (ESD), as well as the curriculum applicable to schools at various levels through guided desk reviews. Group discussions and interview activities were carried out to explore the facilitating factors and obstacles in implementing school health policies related to hygiene, mental health, oral health, nutrition, and ESD during the COVID-19 pandemic. Audio recordings from the interviews and focus group discussions (FGDs) were transcribed verbatim, and thematic analysis was employed to derive the study's findings. The research team ensured that the respondents involved in the study did not have personal relationships with any of the team members. Additionally, to maintain research objectivity, more than one individual was responsible for the coding process (Table 1).

**Table 1:** Themes emerged from deductive analysis

	Content	Process	Actors	Context
1	Lack of detailed guidelines or manual regarding the implementation of school health policy	Lack of realistic comprehensive planning	Inconsistent hierarchy of roles and responsibilities	Decentralisation
2		Integration of CSH policy into the teaching and learning activities	Teachers as role model	
3		Regular healthy school competition program	Existing coordination with relevant stakeholders, school committee participation, and community Engagements	
4		Importance of resources such as funding for consistent CSH implementation	Schools are authorised in making needed-adjustments	

## Results

### Phase #1: the foundation of CSH in Indonesia

Prior to the pandemic, Indonesia had already set up a framework for CSH via the UKS programme started by the Ministry of Education and Culture and the Ministry of Health. With the use of cleanliness practices, dietary counselling, mental health services, and healthcare education, this programme seeks to improve the health of its students. The purpose of these policies is to provide Indonesian students a healthy learning environment.

The school system in Indonesia faced immediate issues following the onset of COVID-19. Essential elements of CSH, such in-person health teaching, school nutrition programmes, and mental health care, were severely disrupted by the closing of schools. To maintain students’ health and well-being while adjusting to a new reality, it is imperative that relevant policies and guidelines be regulated.

### Lack of detailed guidelines or a manual on the implementation of school health policy

Because the regulation only covers a few activities, it lacks comprehensive guidance on implementation of the national school health policy that is now in place. The Indonesian government, for example, has shown its ability to quickly respond to changing circumstances through two regulations: the Guidelines for Developing Sanitation in Elementary School and Management of Clean and Healthy School in Limited Face-to-face Learning. These regulations also highlight the goal of school health initiatives, the recognition of the pandemic as a major challenge, and the involvement of multiple stakeholders. The regulations, however, have certain restrictions. For example, they solely govern the head teacher’s position and the role(s) of the person(s) in charge of activities related to cleanliness. As a result, there was no comprehensive manual for implementing guidelines by both field stakeholders (village level) and supervising stakeholders (district and prefecture level). Moreover, the administration of a clean and healthy school with little in-person instruction only covered the fundamentals of infection control, such as the use of face masks and the placement of hand washing stations. The instructions contained no explicit information or guidelines for changing additional school-specific activities to accommodate the new circumstances. Because there were no clear rules, particularly in the early stages of the pandemic, schools were thus obliged to create their own protocols. Furthermore, there are very few area-adapted regulations pertaining to the execution of school health policies, which is indicative of the wide range of conditions and resources available in each region of Indonesia. Therefore, the currently implemented school-health-related activities merely rely on the initiative of each school to implement them.

“There is some confusion (regarding regulations)... For example, some are conveyed by parents (and) others, for example (information) regulations circulating on social media”

### Lack of realistic comprehensive planning

National laws were designed to make it easy for schools in every region to adopt them. However, the absence of comprehensive implementation standards has led to a lack of thorough preparation. Furthermore, it appears that regulations at the national level are not tailored to the unique peculiarities of the various regions, and as a result, each region must create its own legislation to meet its own requirements. The application of regulations has been uneven due to regional variations in physical and human development and to the possibility that some regions have distinct qualities that set them apart from one another. The Joint Regulation of the Ministers of Education and Culture, Health, Religious Affairs, and Internal Affairs of the Republic of Indonesia Number 6/X/PB/2014 Number 73/2014 Number 41/2014 about the Development of the School Health Unit in Public Schools and Madrasah may be quite clear about who is involved in the school health programme, but it does not go into specifics about their roles. The methods for allocating and guaranteeing appropriate funding while preserving programme viability and how the Central Government will undertake an evaluation that is uniformly applied to each province also remain unclear.

“Even though parents may say, just open schools, but if there are no regulations from the Education Office, yes, we don't dare either.”

### Regular healthy school competition programme

The local health office has been regularly holding school health competitions in accordance with other relevant stakeholders, such as the offices of education and religious affairs. Ensuring school engagement in the implementation of CSH policy and the sustainability of school health programmes that the schools have championed are of utmost importance. Additionally, a few key informants mentioned that they frequently take part in the school health competition programme.

The Minister of Environment of the Republic of Indonesia No. 05/2013 and the Minister of Environment and Forestry of the Republic of Indonesia No. P.53/MENLHK/SETJEN/KUM.1/9/2019 are two examples of the regulations that govern the Healthy School Competition Programme. They both show that the competition will be widely and consistently implemented across all school levels and types. Important information, such as financial support for rival institutions and the methodology and personnel involved in the review, are omitted from these regulations. Additionally, the description of how to guarantee sustainable execution of the school health programme outside of the competition period is somewhat ambiguous.

### Phase #2: immediate response to COVID-19 pandemic

The safety and welfare of all schoolchildren were given top priority by the government during periods when the disease was spreading rapidly. The standards also encouraged a fun, engaging, inclusive, future-focused, and student-capable learning environment. Indonesia responded swiftly with several immediate actions:

1. **Digital learning transition**: Schools embraced digital learning platforms to ensure the continuity of academic and health education. This transition allowed students to access CSH information remotely to promote health awareness. Teachers were also encouraged by governments to improve their teaching approach to keep up with the transition.
2. **Digital mental health support**: Telehealth and remote counselling services were introduced to address the growing mental health concerns in the community and among the students to ensure that they received support despite school closures.
3. **Adaptive nutrition programmes**: School meal services were restructured to provide safer food packages and preparation to ensure that students’ nutritional needs were met.

As the pandemic evolved, Indonesia implemented substantial changes in its CSH policies:

1. **Incorporating digital health education**: CSH policies were updated to include digital health education modules focusing on pandemic prevention, personal hygiene, and mental health resilience. These modules became integral to the remote curriculum.
2. **Strengthening mental health services**: Acknowledging the emotional toll of the pandemic on students, policies were aimed at bolstering mental health services. Virtual counselling and resources were made available to support students’ mental well-being.
3. **Community collaboration**: Policymakers actively encouraged collaboration with local health authorities, parents, and communities to ensure a holistic approach to student health, especially during remote learning.

The government released guidelines that advised local education authorities to coordinate and report to the Ministry of Education and Culture during remote learning to facilitate a fulfilling relationship. They also had to assist in establishing and facilitating rules to provide a supportive learning environment during COVID-19.

### Integration of CSH policy into teaching and learning activities

One important aspect of CSH implementation in Indonesia has been incorporation of the CSH policy into teaching and learning activities. Additionally, some CSH features have been integrated into the national school curriculum. Collaboration with the school health committee, which promotes the integration of CSH into teaching and learning activities, is outlined in the Ministry of Education and Culture Regulation No. 6/2021 and the Regulation of the Secretary General of the Ministry of Education, Culture, Research, and Technology Number 19/2021. Nevertheless, no teacher’s manual or guideline was available for incorporating CSH into the instructional activities. This may have put the initiative’s integration process in danger and confused the initiative’s stakeholders.

“During the Covid period, we requested one of the parents who is a specialist in pulmonary medicine to present an explanation about what Covid is to the children.”

### Inconsistent hierarchy of roles and responsibilities

One thing that impeded the implementation of CSH was the absence of specific instructions for the work of officers at the provincial and district levels in each prefecture. The policies were not being executed as they should be because it was still unclear what the district level officers’ responsibilities were when conducting the given policies and guidelines. The desired outcomes of the regulation’s execution were not ideally attained due to the error in role assignments. Additionally, the policies’ lack of clarity made their implementation difficult, which resulted in the policies in place being ineffective.

For instance, the social welfare sector and community health centres/workers participated in the implementation of school health programmes according to the Minister of Health’s Verdict Nos. HK.01.07/MENKES/382/2020 and HK.01.07/MENKES/413/2020. To address the COVID-19 pandemic in Indonesia, the regulations also laid forth the preventative and control measures to be taken. Nonetheless, certain crucial information was still missing, such identifying the participants and their responsibilities and the person in charge of each area or location. Consequently, it was usually unclear as to whose recommendations should be followed.

“…because those who are competent for this are physical education teachers, so they are the ones who handle issues related to health.”

” I (the Principal) submit the report to the education office, as well as to the community health centre (Puskesmas), because every week their staff certainly visits the school. The principal or the class guardian is usually the one being questioned.”

### Phase #2: ensuring safety and learning continuity in school reopening

As Indonesia worked toward safely reopening schools, the nation’s policies addressed the following:

1. **Hybrid learning models**: Hybrid models combining in-person and remote education were introduced that adhered to strict health guidelines to ensure the safety of students and staff. This involved assuring the availability of disinfection fluid, masks, thermal guns, and health monitoring.
2. **Vaccination strategies**: Comprehensive strategies were formulated to ensure the efficient distribution of vaccines to school staff and eligible students to minimise the risk of outbreaks within educational settings.
3. **Continued mental health focus**: In recognition of the lingering emotional impact of the pandemic, mental health support remained a priority as students returned to schools. Thus, schools provided easy access to mental health service for all school members including those confirmed to have COVID-19.
4. **Preparedness for school reopening**: All educational institutions were obliged to inform the government of their preparations and readiness for direct learning. In accordance with this information, the government had to help ensure the readiness of the schools. Based on the government’s regulation, in-person learning was not allowed in dangerous zones designated as orange or red. School reopening could not be undertaken if the requirements were not fulfilled.
5. **Responsibility of education authorities**: A safe place based on health care guidelines needed to be provided. The head of the education unit had to monitor and report school residents who were prohibited from conducting activities during school reopening. If there were confirmed cases of COVID-19 among school attendees, the government could use its authority to close the school temporarily. Treatment and medication reports also had to be delivered as soon as possible.
6. **Direct learning mechanism**: Disease transmission is a problem that must be prevented as much as possible. Therefore, heads of education units were charged with arranging direct learning mechanisms such as organising the division of learning groups with appropriate schedule settings, arranging seating distance for each school member to limit crowding, and ensuring sufficient air circulation.
7. **Prohibition of non-academic activities**: Sports, extracurricular activities, and parent meetings were prohibited due to the potential for COVID-19 transmission. School canteens were prohibited from operating, and all school members were suggested to bring their own food.

### Importance of resources such as funding for consistent CSH implementation

Facilities and funding are two crucial elements that affect how well CSH is implemented. Nonetheless, many of the schools voiced their worries about not having enough funding to maintain school health programmes and ensure that essential buildings and services, such as the UKS, remained operational and representative. For example, mobile data support for students to participate in online learning activities was addressed by regulations such as the Ministry of Education and Culture Regulation No. 6/2021 about Technical Management Regular School Operational Assistance Fund and the Secretary General of the Ministry of Education and Culture Regulation Number 4/2021 concerning Technical Guidelines for the Distribution of Government Assistance Internet Data Quota Package in 2021. However, these laws do not specify how mobile devices will be paid for or how they will assist students who live in places without internet access. To determine how to distribute funds to children who are financially disadvantaged because of COVID-19 infection, the regulations must also specify how monitoring and evaluation procedures should be conducted. Additionally, some schools rely on donations from parents or self-funding to ensure the proper implementation of CSH programmes. For instance, to compete in the school health competition, schools must improve the quantity and quality of physical facilities such as restrooms and waste bins. As well, to properly run a UKS, adequate availability of over-the-counter medications is necessary as are regular health training and workshops for teachers and students.

” The school organizes it independently, sometimes there are parents who voluntarily contribute.”

### Teachers as role models

Teachers have multiple responsibilities when implementing CSH in schools. They start by establishing a welcoming environment for students, which includes not only keeping the school cosy, but also stressing healthy behaviours and modelling clean and healthy living habits for each student. Handwashing before entering class and before eating and maintaining school grounds and classrooms are examples of how children might become used to practising healthy and clean-living habits. Following an explanation of the value of maintaining cleanliness to ward off illness, teachers demonstrate how to wash hands correctly with soap or hand sanitizers. Additional events held at the school include the recurring "gotong royong" which is an Indonesian term meaning working together and the sorting of hazardous, non-hazardous, and organic rubbish to preserve environmental cleanliness.

In relation to the pandemic, the Joint Decree of the Minister of Education and Culture, the Minister of Religious Affairs, the Minister of Health, and the Minister of Internal Affairs of the Republic of Indonesia No. 03/KB/2021, No. 384/2021, No. HK.01.08/MENKES/4242ENG/2021, and No. 440–717/2021 and the Joint Decree of the Minister of Education and Culture, the Minister of Religious Affairs, the Minister of Health, and the Minister of Internal Affairs of the Republic of Indonesia No. 01/KB/2020, No. 516/2020, No. HK.03.01/Menkes/363/2020, and No. 440–882/2020 convey the technical details on restricted face-to-face learning. Teachers were positioned as the primary role models for students in its implementation and were urged to maintain discipline, arrive at school early, wear face masks and wash their hands properly, avoid littering, and refrain from using derogatory language or acting violently towards students or one another. Particularly, younger students frequently pay more attention to and sometimes have higher regard for their teachers than for their own parents. A lot depends on each teacher’s individual personality and public demeanour. Instructors have the power to influence pupils who engage in harmful activities, including smoking, provided that the behaviour is observed by the students and takes place near the school.

“With direct practice, for example, if you punish a child for being late, then what should be done is to remind them not to be late. There I have explained to them that if you punish a child for not completing their homework, as a teacher, you must also start by doing so yourself.”

“Appreciation for the teachers, they are also human, they also have fears, they also have health concerns, meaning they are indeed human, but they are brave and willing to dedicate themselves, they teach not from home, they teach at school. Therefore, they also pay attention to how to prevent transmission.”

### Phase #3: Recent development and future prospects

Recently, Indonesia has shifted its focus to the following:

1. **Long-term resilience**: Policies now emphasise building long-term resilience in schools through an increased focus on disaster preparedness and improved health infrastructure.
2. **Addressing learning loss**: Strategies to mitigate learning loss and address educational disparities stemming from the pandemic are being incorporated into CSH policies to ensure that no student is left behind.
3. **Digital equity**: Policymakers are working diligently to bridge the digital divide to ensure equitable access to online resources and technology.

#### Schools are authorised to make needed adjustments

In Indonesia in particular, the COVID-19 pandemic has affected the educational system. Consequently, the Ministry of Education and Culture released Circular Letter No. 4/2020, which governs the Policies for Education Implementation during the Emergency Phase to Stop the Coronavirus Disease (COVID-19) from Spreading. Instruction on how to transition from in-class activities to remote or home-based learning is included in this regulation. The principal of the school also has the power to enforce the regulation, including providing guidance and inspiration to instructors so they can facilitate online learning throughout the pandemic. Parental and school staff communication on the changes to the learning process was done via a WhatsApp group. Schools can also adjust the implementation of face-to-face learning trials to ensure a safe duration of contact. However, this has also led to differences in CSH implementation between schools.

”…as there are differences before and after COVID. Before COVID, we had the freedom to go anywhere, whether wearing a mask or not. Now, when we implement the program, the most important aspect is to emphasize health protocols first. For instance, the junior high school is planning an outdoor camping program for the children, which was actually planned three years ago, but it is only this year that we will roll it out again. Of course, the consequence is that there will be a noticeable difference between the program before COVID and the current one.”

#### Existing coordination with relevant stakeholders, school committee participation, and community engagements

To assess the success of the CSH implementation and set goals for its accomplishment, the school and its stakeholders—Primary Health Centres, or Puskesmas, Local Health Offices, the National Agency on Narcotics (Badan Narkotika Nasional), and the Food and Drug Supervisory Agency (Badan Pengawas Obat dan Makanan)—cooperate in managing the UKS. The initiatives include monitoring school cafeterias, promoting awareness of reproductive health, and preventing drug use and addiction. Subsequent enhancements and evaluations of the degree of benefits derived from the implemented CSH programme, if accompanied by appropriate ongoing oversight of the planning and execution phases, would be able to identify obstacles that occur at every stage of the implementation.

Some of the regulations that support this theme include No. 04/KB/2020, No. 737/2020, No. HK.01.08/Menkes/7093/2020, No. 420–3987/2020, and the Minister of Health Verdict No. 1429/MENKES/SK/XII/2006. These are Joint Decrees of the Ministers of Education and Culture, Religious Affairs, Health, and Internal Affairs of the Republic of Indonesia. These laws specify the current cooperation in the fight against the pandemic amongst several ministers, school administrators, and community health centres. Distance learning is also supported and ensured by coordination amongst many ministries. Nevertheless, while long-term and sustainable coordination is ideal, this law does not specify how to keep track of or assess the multiple parties involved in coordination, which could lead to low retention. Moreover, in relation to CSH, school mapping should ideally be done to create a school policy by involving multiple stakeholders such as teachers, school staffs, and local residents. However, this step is not specified in the regulations.

“ … coordinating with the military because we will be implementing extracurricular activities (Little Doctors or Red Cross), which also important for the students.”

“Because the committee is also a channel for the representatives of the parents, the committee's statement will certainly be a summary of the results of the parents' opinions and aspirations, which we will refine if there is a need for improvement related to perhaps the SOP regarding COVID prevention and so on that needs to be sharpened.”

#### Decentralisation

Although decentralisation or regional autonomy first came into force in 1999, it was already acknowledged since the very beginning of Indonesia’s sovereignty and even stated in the 1945 constitution. The strategy is a factor that influences the perception of national-level officers that regional officers have more autonomy and responsibility for the implementation of policies including the comprehensive school health policy. Decentralisation enables regional officers to have more control in the implementation of the policy in their particular district, hence providing a better understanding and adaptation of such policy with the actual condition. A context-based approach such as the Open School programme that was designed to address high school dropout rate in WNT. This programme is suitable to be implemented in WNT because school drop outs due to child marriage and other heavily embedded local customs are still commonly found.

Although national-level regulations that regulate autonomy of regional authority on health and education exist, the regulations issued by local governments are not widely accessible. This has caused difficulties to confirm regulations referred by the implemented specific CSH programmes designed to suit the regional needs. Moreover, this approach would vary from one region to another. Although evidence is still lacking in terms of its effect on the implementation of school health policy, a more context-appropriate approach would inevitably suit the needs of the local people better than a more generalised non-contextualised approach.

The differences due to geographic, socioeconomic, and cultural barriers are not always easily bridged, causing one approach can be more accommodating the needs of certain specific groups of people but not the other. Moreover, in WNT specifically, the role of religious and community leaders is quite prominent. Programmes implemented at schools are often by cooperating with religious and community leaders, hence, the programmes have to be socially and culturally acceptable. Therefore, there is consequently a need for more empirical studies to document the likely effects of decentralisation in the implementation of school health policy.

## Discussion

The goal of school health efforts is to enhance students’ and staff members’ capacity to lead healthy lives and to adopt clean, healthy lifestyles. Therefore, the goal of the school health initiative is to help students live healthier lives [9]. Students are motivated to maintain and improve their health, protect themselves from diseases, and cut back on risky behaviours because schools are among the places where students’ behaviour is formed. As a result, campaigns to promote clean and healthy living habits, maternal and child health, prevention of infectious diseases, and vaccinations, among others, can be held there. The way the children behave and interact with others in the community will then be determined by their developed behaviour [10].

A few examples of the variables that affect school health initiatives are collaborations with healthcare providers, curriculum, school organisation, and individual ethos. Thus, for school health initiatives to be successful, schools must consider health issues that arise in the school and surrounding communities, organisational capacities, including knowledge and skills, dedication, organisational structure, and leadership elements. Incorporating health promotion into the curriculum and teaching materials, along with receiving support from administrators, teachers, and other school staff members, are additional crucial components in boosting school health and health education. Additional necessities include the availability of consistent funding, spaces for health promotion initiatives, and collaboration with relevant stakeholders.

Comprehensive strategic and operational planning at the federal, state, local, or even educational levels ought to be the initial stage of the distribution of information and its execution. Thorough planning could help with the process of implementing and adjusting policy to align with what is happening in each school. According to data from a pilot programme in Kenya, careful planning allowed for the alignment of the policy’s development, actual execution, and its adaptation to each school’s pre-existing school health programmes [11].

Potential conflicts with the current programme, the Healthy School Competition Programme, may arise from the CSH policy that is now being suggested. Despite this conflict, not every district implements this programme in the same way. Consequently, it is imperative that every district appropriately adjusts to the current programme in that district and vice versa. A new programme based on the CSH policy will need to be developed by stakeholders in the districts in which such a programme is already regularly implemented to further improve its current performance [12].

For children to perform at their best in school, a successfully implemented school health policy should foster an environment in which teaching and effective learning take place in an environment of good health. According to the South African Integrated School Health Policy, school health policies are likely to serve as a safety net for children with no access to preventive care or who cannot recognise health issues that could be preventing them from getting better and impeding their ability to learn and for teachers to teach them [12]. Its incorporation into the teaching and learning process would therefore boost the dissemination and execution of school health policy while also improving the teaching and learning process itself in a synergistic way [13].

To achieve a specific aim, in this case the broad dissemination and ingrained execution of a CSH policy, it is necessary to make specific functions clear to the relevant individual or group. Role clarification also makes it possible for them to participate in capacity-building and training initiatives that may strengthen current positive cultures, foster trust, and broaden their understanding. In addition, defining roles facilitates coordination amongst the key actors. Therefore, ambiguous job clarifications run the risk of confusing those working on the implementation of CSH policies. According to one report, this uncertainty had a detrimental effect on how well the CSH strategy was implemented [14].

One reported barrier to the effective dissemination and application of school health policies was the lack of financial support for these activities [12]. As stated in The Mississippi Healthy Students Act of 2007, sufficient financing is essential for any school health policy or programme to be implemented successfully and enhance students’ health [15]. Physical infrastructure and human resources are strongly reliant on the level of funding provided by relevant parties [16]. Due to funding obstacles, schools can only undertake a small number of the numerous programmes or policies that the government mandates as they have limited resources to implement the policies.

Comprehensive manuals or guidelines are essential for putting school health policies into practice [17]. According to research conducted in 2020, the most important and beneficial aspect in the distribution and application of school health policy is having access to specific manuals or guidelines, whether available in paper copy or electronic format [12]. According to that study, having such a document would help school administrators understand the rationale behind the policy’s execution and plan a methodical rollout that is tailored to their school’s unique working conditions. Because a comprehensive guideline was a key component of a framework that effectively disseminated school health policies in Nigeria, it also helped to raise awareness of this policy among more school principals [13, 17].

Health promotion in schools can be more comprehensive and sustainable if parties collaborate across sectors to promote it. Thus, there is a real need for a collaborative partnership between central and local government, the community, and other relevant institutions for school health policies to be implemented successfully [18]. Advocacy and collaboration, partnership, integration, and consensus building as evidence for the dissemination and implementation of a policy on school health are perceived as positive factors [12, 13, 16]. Dissemination and implementation of a school health policy are influenced by a context wherein implementation is conducted by the actors, in this case stakeholders, who are actually disseminating and implementing it [19]. Cooperation between schools and the community also play a crucial role in increasing the awareness of such policies, which indicates the importance of community engagement [16, 17].

Considering their authority in implementing policy, school principals therefore function as the main stakeholder in the implementation of school health. Practically, they are a government’s last frontier before the regulations or guidelines are executed by teachers, person in charge, or even students. Within the reviewed regulations, the scope of a school principal’s responsibilities in regard to the implementation of CSH encompasses coordinating the adaptation process of such regulations to the actual condition in their respective schools, managing the budget of such implementation, performing monitoring and evaluation of such policy, and then fine-tuning it to create a more suitable implementation for their particular conditions. These regulations also illustrate the variety of sectors being managed by the school principal.

In actually executing the programmes or policies involved in the implementation of CSH, every programme in the school health implementation strategies or guidelines will involve teachers one way or the other, be it as a supervisor, person in charge, or even the actual target of the programmes or policies. Therefore, it is safe to assume that teachers are deeply involved in promoting school health. This involvement is also applied the other way around. In the implementation process, any feedback obtained from all sources such as students, community, parents, or even other teachers will be directly or indirectly received by the teachers in hopes of them improving it.

However, the integral part being played by teachers in promoting school health also poses a threat to school health implementation. From the interviewees, key informants, and school observations, we found a lack of monitoring and evaluation of the implementation of school health and also differences in implementation that were caused by a disparity in the resources, in this case human resources, possessed by the different schools. This condition can potentially jeopardise the consistent implementation of school health.

Community-based approaches are one means of supporting comprehensive and sustainable school health. When communities are involved in improving school health, health promotion programme can become more specific and tailored to the health problems that each community faces. This is consistent with the school health promotion model proposed by WHO in 1997, which recognises that teaching staff, students, and the community all play significant roles in promoting school health [20–22].

One of the efforts to promote school health with a community approach is to conduct activities that can guarantee the creation of a clean, healthy, and adequate school and surrounding environment. Communication and collaboration between the school and parents regarding the focus of health promotion in schools such as implementing clean and healthy-living behaviours and good diet patterns are important. Good cooperation between schools and parents will increase the chances of students becoming obedient in carrying out the health promotion techniques that have been taught. In Indonesia itself, the government has provided school health support facilities with a community approach through the UKS. The UKS programme is expected to improve the health status of students through more focused and comprehensive health promotion, especially related to Clean and Healthy Life Behavioral programme with assistance from nurses or other health workers at the local public health clinic [20–22].

Support from the community will make or break the sustainability of any programme or policies of school health that are being implemented. Good and adequate support will enhance the sustainability of anything that the students obtain from any policy or programme, whereas the lack of it will easily hinder the actual objective of CSH, which is to promote health and wellness and prevent high-risk social behaviours. Thus, if the community has perfectly played their role in the implementation of school health, anything that the children obtain from school health implementation during school time will be further integrated into their life because there will be no disparities between what they told to do and how to behave in school and how the community actually behaves. However, disparities can jeopardise the sustainability of such implementation as we found in the interviews and school observations, which showed that a disparity between school and community indicating a socioeconomic and cultural barrier will lead to diverse understanding of the concept of school health. In turn, this will act to hinder the implementation of CSH.

## Conclusion

Indonesia's CSH policies have demonstrated flexibility and dedication to the welfare of its students during the COVID-19 pandemic. The country's focus on long-term resilience and equity has evolved significantly, despite initial difficulties in adjusting to online learning and distant counselling. The dynamic development of CSH policies in Indonesia demonstrates the country's commitment to fostering students' holistic well-being. These policies have remained at the forefront, providing a safe, resilient, and healthy educational environment for Indonesia's students, even as the country navigates the challenges brought on by the pandemic. The nation's commitment to promoting the holistic well-being of its students is evident in the dynamic evolution of CSH regulations in Indonesia.

Preventing the spread of SARS-CoV-2 virus was accomplished by implementing travel restrictions, lockdown, and isolation. Although these actions are believed to be helpful in lowering the risk of transmission, other aspects, such the effects on socioeconomic status and education, were considerably diminished. The Indonesian government coherently introduced an initiative aimed at reducing the risk of viruses spreading through in-person instruction and learning activities by closing schools. Teaching and learning procedures were changed to overcome this problem and allow children to continue learning during the pandemic. Our policy review found an interesting dynamic of the CSH policy before the COVID-19 pandemic, the nation’s immediate response to the pandemic, and future prospects as a consequence of the pandemic.

Indonesia already had a CSH structure and programmes in place even before the pandemic began that were intended to provide a healthy learning environment for Indonesian students. However, as the nature of the COVID-19 pandemic was unheralded, no policy was able to completely mitigate its impact on the education process. The main aspect that can be further improved by all related stakeholders in this phase is for Indonesia to develop more realistic, detailed, and comprehensive guidelines for the implementation of school health policy. In the immediate response to the pandemic, the rapid policy-making process resulted in an inconsistent hierarchy of roles and responsibilities regarding the implementation of CSH. Therefore, a dedicated policy regarding sustainable cooperation and coordination between related stakeholders during an unprecedented event in the future is needed to produce a better policy if such an event occurs again. More detailed and thorough policies should also be introduced to ensure consistent implementation. Indonesia should also more greatly emphasise its distinctive decentralisation by giving more authority to local government to implement necessary actions to maintain the standard delivery of teaching and learning activities.

As Indonesia continuous to navigate the ongoing challenges posed by the pandemic, the effective implementation of CSH policies remains paramount. These policies not only address immediate health concerns, but also prioritise the overall well-being of the students, thereby ensuring that education remains accessible and supportive. Moving forward, it is essential for stakeholders to build on this foundational commitment by developing comprehensive guidelines that facilitated sustainable cooperation among relevant parties and empower local governments to implement necessary actions. Through these efforts, Indonesia can enhance its educational framework and better prepare for future challenges.

## Data Availability

The data that support the findings of this study are available from Department of Global Health, Graduate School of Health Sciences, University of the Ryukyus and The Faculty of Medicine and Health Sciences, University of Mataram. Restrictions apply to the availability of these data, so are not publicly available. The data are, however, available from the authors upon reasonable request and with the permission of Department of Global Health, Graduate School of Health Sciences, University of the Ryukyus and The Faculty of Medicine and Health Sciences, University of Mataram.

## Abbreviations

COVID-19: Coronavirus disease 2019
CSH: Comprehensive school health
SARS-CoV-2: Severe acute respiratory syndrome coronavirus 2
UKS: Unit Kesehatan Sekolah/School Health Unit
WHO: World Health Organization
